# Robotic-Assisted vs. Open Simple Prostatectomy for Large Prostates: A Meta-Analysis

**DOI:** 10.3389/fsurg.2021.695318

**Published:** 2021-07-20

**Authors:** Zhongyou Xia, Jinze Li, Xiaoying Yang, Hao Jing, Chao Niu, Xianhui Li, Yunxiang Li, Zongping Zhang, Ji Wu

**Affiliations:** ^1^Department of Urology, Nanchong Central Hospital, The Second Clinical College, North Sichuan Medical College, Nanchong, China; ^2^Department of Urology, Institute of Urology, West China Hospital, Sichuan University, Chengdu, China; ^3^West China School of Medicine, Sichuan University, Chengdu, China; ^4^Blood Purification Center of Department of Nephrology, Nanchong Central Hospital, The Second Clinical College, North Sichuan Medical College, Nanchong, China; ^5^Department of Urology, Pidu District People's Hospital, Chengdu, China

**Keywords:** benign prostatic hyperplasia, simple prostatectomy, robotic, open, meta-analysis

## Abstract

**Purpose:** To compare the efficacy and safety of robotic-assisted simple prostatectomy and open simple prostatectomy for large benign prostatic hyperplasia.

**Methods:** We systematically searched the Cochrane Library, PubMed, Embase, and Science databases for studies published through December 2020. Controlled trials on RASP and OSP for large prostates were included. The meta-analysis was conducted with the Review Manager 5.4 software.

**Results:** A total of seven studies with 3,777 patients were included in the analysis. There were no significant differences in IPSS (WMD, 0.72; 95%CI: −0.31, 1.76; *P* = 0.17), QoL (WMD, 0.00; 95%CI: −0.39, 0.39; *P* > 0.99), Qmax (WMD, 1.88; 95% CI: −1.15, 4.91; *P* = 0.22), or PVR (WMD, −10.48; 95%CI: −25.13, 4.17; *P* = 0.16) among patients undergoing RASP and OSP. However, compared with patients who underwent OSP, patients who underwent RASP had a shorter LOS (WMD, −2.83; 95%CI: −3.68, −1.98; *P* < 0.001), less EBL (WMD, −304.68; 95% CI: −432.91, −176.44; *P* < 0.001), a shorter CT (WMD, −2.61; 95%CI: −3.94, −1.29; *P* < 0.001), and fewer overall complications (OR, 0.30; 95% CI: 0.16, 0.57; *P* < 0.001). Nevertheless, RASP was associated with a longer OT (WMD, 59.69, 95% CI: 49.40, 69.98; *P* < 0.001).

**Conclusion:** The results of the current study demonstrated that RASP provided similar efficacy to those of OSP in the treatment of large prostate, while maintaining better security. Our findings indicate that RASP is a feasible and effective alternative to OSP.

## Introduction

Benign prostatic hyperplasia (BPH) and its associated symptomatology affect many men worldwide; as of 2010, over 210 million men had been diagnosed with BPH. Moreover, 50% of men older than 50 years and about 80% of men older than 80 years experience lower urinary tract symptoms (LUTS) due to BPH (1). Lifestyle modifications and drug therapy are generally the first-line treatment for symptomatic BPH (2). However, BPH with enlarged prostate (>80 mL) and severe symptoms is associated with poor drug therapy and disease progression. According to the American Urological Association guidelines, surgery is recommended for patients who experience renal insufficiency, recurrent urinary tract infections, bladder stones, or gross hematuria due to BPH and those who have LUTS refractory to other therapies and/or persistent LUTS under medical management. Transurethral resection of the prostate has been considered the gold standard for the surgical treatment of BPH; however, it is limited by the volume of the prostate and resection time. Therefore, endoscopic resection or simple prostatectomy have become the main management options for these patients (3, 4).

However, surgery for BPH weighing 80–100 g or more poses a major challenge for surgeons. Open simple prostatectomy (OSP) has been the standard surgical treatment for men with moderate to severe LUTS and a prostate size larger than 80 mL (3). However, OSP has significant side effects, like bleeding, requirement for blood transfusion, and revision surgery (5). With the development of minimally invasive surgery, laser enucleation and laparoscopic surgery are being more commonly performed in the clinic (6, 7). However, these procedures have a steep learning curve, and laser enucleation can cause long-term transient stress urinary incontinence (SUI) (8). Since Sotelo et al. (9) first performed robot-assisted simple prostatectomy (RASP) in 2008, its safety and effectiveness have been recognized. Therefore, RASP is thought to be a minimally invasive alternative to OSP. However, RASP and OSP in the treatment of large BPH remain controversial (9, 10).

Therefore, we performed a systematic review and meta-analysis of the efficacy and safety between RASP and OSP for large glands in the contemporary robotic era to provide a better clinical reference.

## Methods

### Search Strategy

We systematically searched the Cochrane Library, PubMed, Embase, and Science databases from inception through December 2020. We used the following search terms: “prostatic hyperplasia,” “BPH,” “benign prostatic hyperplasia,” “open prostatectomy,” “open adenomectomy,” “robot,” and “robotic surgery.” Search strategies were tailored for the different search engines. Further, a complete manual search of the references in the relevant articles, as well as the minutes and abstracts, was performed. The search was not limited by region or language. Two researchers independently conducted preliminary screening, evaluation, and data extraction of the literature.

### Study Selection

All eligible studies were enrolled in the meta-analysis based on pre-designed inclusion and exclusion criteria. The inclusion criteria were as follows: (1) patients diagnosed with BPH; (2) comparative analysis of RASP and OSP for treating BPH of at least 80 mL or 80 g; and (3) at least one corresponding outcome indicator. The exclusion criteria were as follows: (1) prostate cancer patients; (2) patients who underwent previous urinary tract, prostate, or bladder neck surgery; (3) studies on other surgical treatments of BPH; (4) no control group; (5) lack of data comparisons required for meta-analysis; and (6) editorial comments, meeting abstracts, reviews, letters, case reports, or comments.

### Data Extraction

All outcomes of interest were independently extracted by two investigators (Z.X. and J.L.) who solved any differences through discussion. Finally, a senior author (J.W.) resolved all disagreements after public discussion. The extracted data included post-void residual urine volume (PVR), international prostate symptom score (IPSS), maximum urine flow rate (Qmax), quality of life (QoL), operative time (OT), estimated blood loss (EBL), transfusion rate (TR), catheterization time (CT), and length of hospital stay (LOS). The enrolled studies were assessed by one reviewer (X.Y.).

### Quality Assessment

The quality of all included studies was estimated using the Newcastle–Ottawa scale (maximum score 9). A score of ≥6 was considered high quality, whereas a score of ≤ 5 indicated low quality. Two reviewers (Z.X. and J.L.) performed quality assessment and assessed the level of evidence of the included studies according to the Oxford Center for Evidence-based Medicine (Table 1), and differences were resolved through negotiation.

**Table 1:** Basic characteristics and quality assessment of included studies.

**Study**	**Design**	**Intervention**	**Age (years)**	**Prostatevolume(ml)**	**BMI**	**RSAP approach**	**Quality scores**	**LE**
		**RASP(N)/OSP(N)**						
Hoy et al. (11)	Retrospective comparative trial	4/28	69.3 ± 2.9/75.2 ± 6.4	239.0 ± 49.8/180.0 ± 54.7	_	Transperitoneal	7	4
Sorokin et al. (12)	Retrospective comparative trial	64/103	68.8 ± 8.0/68.0 ± 7.5	136.2 ± 46.6/147.3 ± 50.1	28.6(±4.5)/29.5(±4.4)	Transperitoneal	6	3b
Mourmouris et al. (10)	Prospective comparative trial	26/15	66.7 ± 8.6/70.5 ± 4.8	>80.0/>80.0	_	Transperitoneal	8	2b
Nestler et al. (13)	Prospective comparative trial	35/35	70.1 ± 5.1/70.3 ± 6.3	104.8 ± 41.7/104.2 ± 37.1	_	_	8	2b
Hamann et al. (14)	Retrospective comparative trial	39/39	73 ± 8.4/74 ± 6.9	130.5 ± 42.2/113.5 ± 28.7	_	Transperitoneal	6	4
Dotzauer et al. (5)	Retrospective comparative trial	24/103	71 ± 7.3/72 ± 6.9	127 ± 32/119 ± 25	27.3 ± 3.2/27.8 ± 4.7	Transperitoneal	6	3b
Bhanvadia et al. (15)	Retrospective comparative trial	704/2,551	67.8 ± 8.0/71.0 ± 8.1	>80.0/>80.0	_	_	7	4

*BMI, body mass index; RASP, robot-assisted simply prostatectomy; OSP, open simple prostatectomy; LE, level of evidence according to the Oxford Center for Evidence-based Medicine*.

### Statistical Analysis

The Cochrane Collaborative RevMan5.4 software was used for meta-analysis statistical processing in our study. The weighted mean differences (WMDs) and odds ratio (ORs) were calculated for continuous and dichotomous variables, respectively, with 95% confidence intervals (CIs). χ^2^ test and *I*^2^ test were used to analyze the heterogeneity between the studies. If there was significant heterogeneity (*p* < 0.05 or *I*^2^ > 50%), a random-effects model was used; otherwise, a fixed-effects model was adopted.

## Results

### Study Characteristics

**Figure 1:**
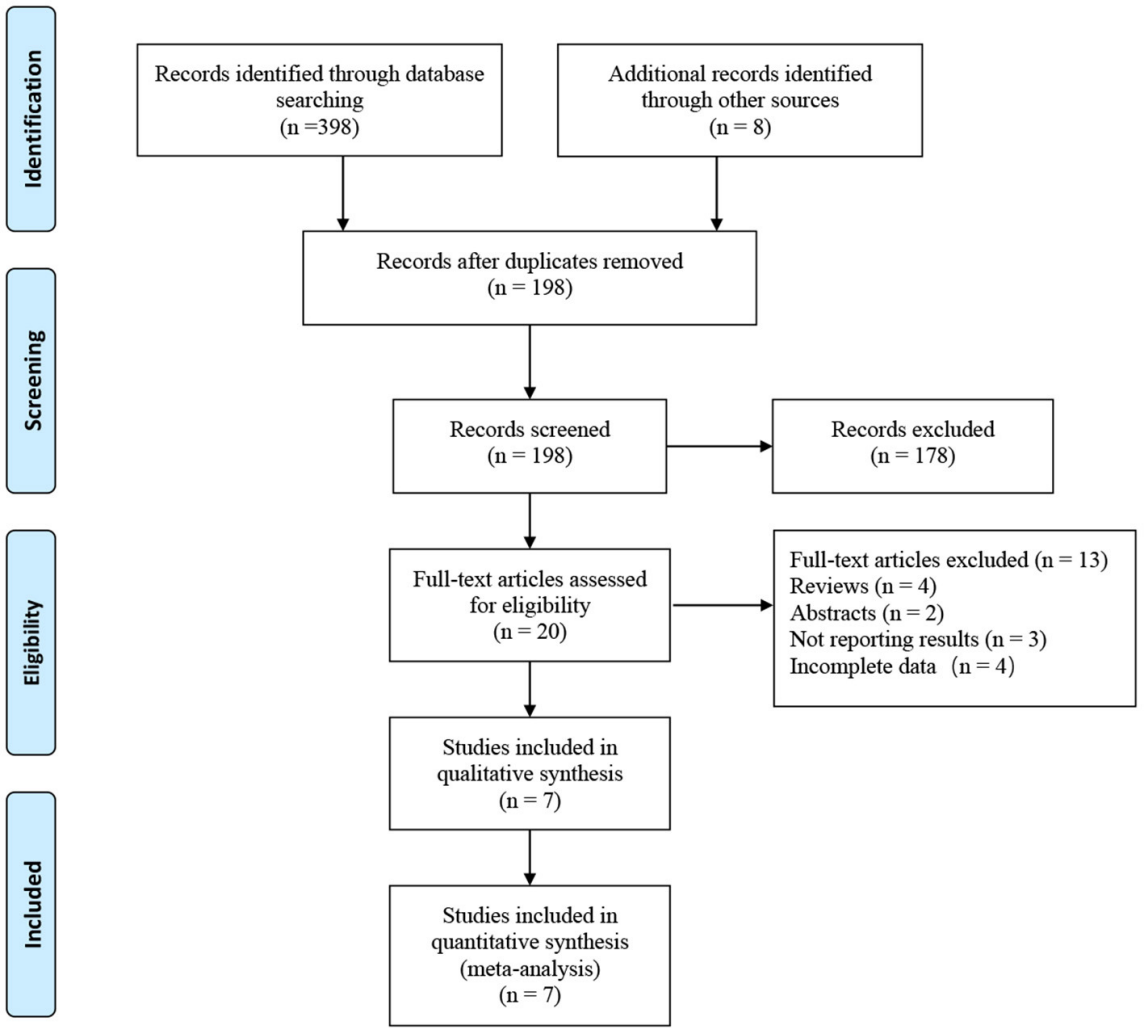
Flow diagram of PRISMA.

A total of 406 related articles were preliminarily detected. Among them, 208 records were excluded because of duplication or because they were irrelevant to our inclusion criteria based on the screening records. One hundred and seventy-eight records were removed after review of the full text. Finally, the remaining seven studies with 3,777 patients (975 in the RASP groups and in the 2,802 group) were included in our meta-analysis (Figure 1) (5, 10–15). The quality evaluation of the included studies is presented in Table 1. Table 2 displays the pre-operative parameters of included studies in the meta-analysis.

**Table 2:** Pre-operative parameters of included studies in the meta-analysis.

**Study**	**OSP**					**RASP**				
	**IPSS**	**PVR**	**Qmax**	**PSA**	**QoL**	**IPSS**	**PVR**	**Qmax**	**PSA**	**QoL**
Hoy et al. (11)	_	_	_	_	_	_	_	_	_	_
Sorokin et al. (12)	18.2 ± 6.5	152 ± 148.1	8.9 ± 5.0	7.4 ± 4.8	3.9 ± 1.5	18.4 ± 8.1	164.3 ± 111.5	10.1 ± 6.8	7.2 ± 5.8	3.9 ± 1.4
Mourmouris et al. (10)	23.54 ± 5.34	246.5 ± 252.23	9.10 ± 3.11	11.34 ± 15.12	_	22.87 ± 4.33	178.50 ± 190.54	10.11 ± 2.66	10.82 ± 12.32	_
Nestler et al. (13)	23 ± 3.09	_	_	_	5.33 ± 0.77	22.7 ± 3.86	_	_	_	5 ± 1.55
Hamann et al. (14)	_	_	_	10.7 ± 10.0	_	_	_	_	7.7 ± 5.2	_
Dotzauer et al. (5)	17.0 ± 6.6	180 ± 176	16.4 ± 16.8	_	_	17.3 ± 7.4	185 ± 183	6.1 ± 3.8	_	_
Bhanvadia et al. (15)	_	_	_	_	_	_	_-	_	_	_

*IPSS, International Prostate Symptom Score; PVR, post-void residual urine volume; Qmax, maximum urinary flow rate; PSA, prostate specific antigen; QoL, quality of life*.

### Surgical Outcomes

#### Operating Time

**Figure 2:**
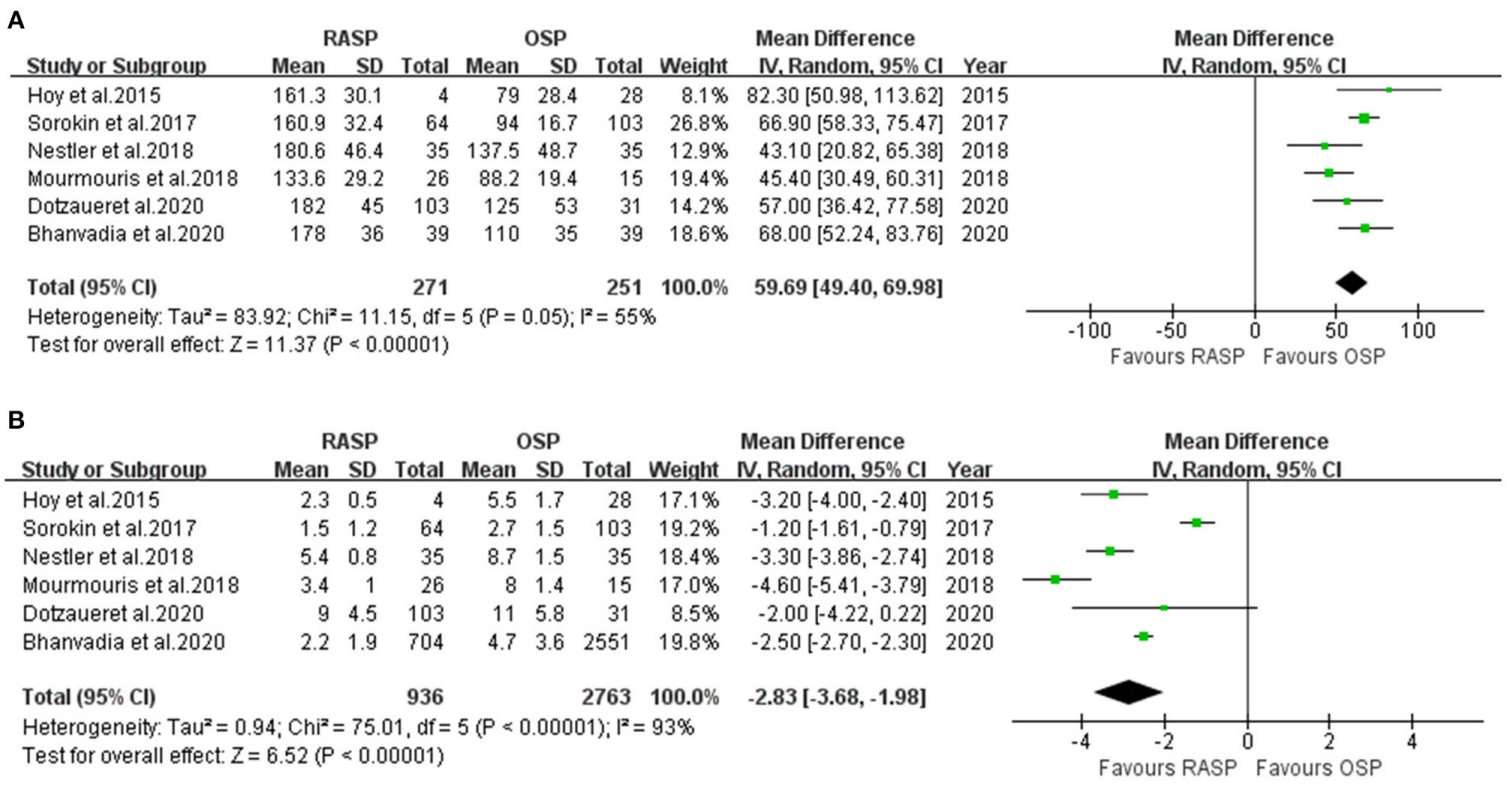
Forest plot and meta-analysis of OT **(A)** and LOS **(B)**.

Five studies including 522 patients were included in the meta-analysis of OT. Among them, 271 underwent RASP and 251 underwent OSP (Figure 2A). Because the heterogeneity was high, a random-effects model was used (*I*^2^ = 55%). The pooled outcome supported that OT was longer in the RASP group than in the OSP group (WMD, 59.69; 95% CI, 49.40, 69.98; *P* < 0.001).

#### Length of Hospital Stay

LOS data were reported in six studies involving 3,699 patients (Figure 2B) (5, 10, 12–15), of whom 936 underwent RASP and 2,763 underwent OSP. Patients treated with RASP had a shorter LOS. There was a statistically significant difference between the two groups in LOS (random-effects model: WMD, −2.83; 95% CI, −3.68, −1.98; *P* < 0.001; *I*^2^ = 93%), despite high heterogeneity.

#### Estimated Blood Loss

**Figure 3:**
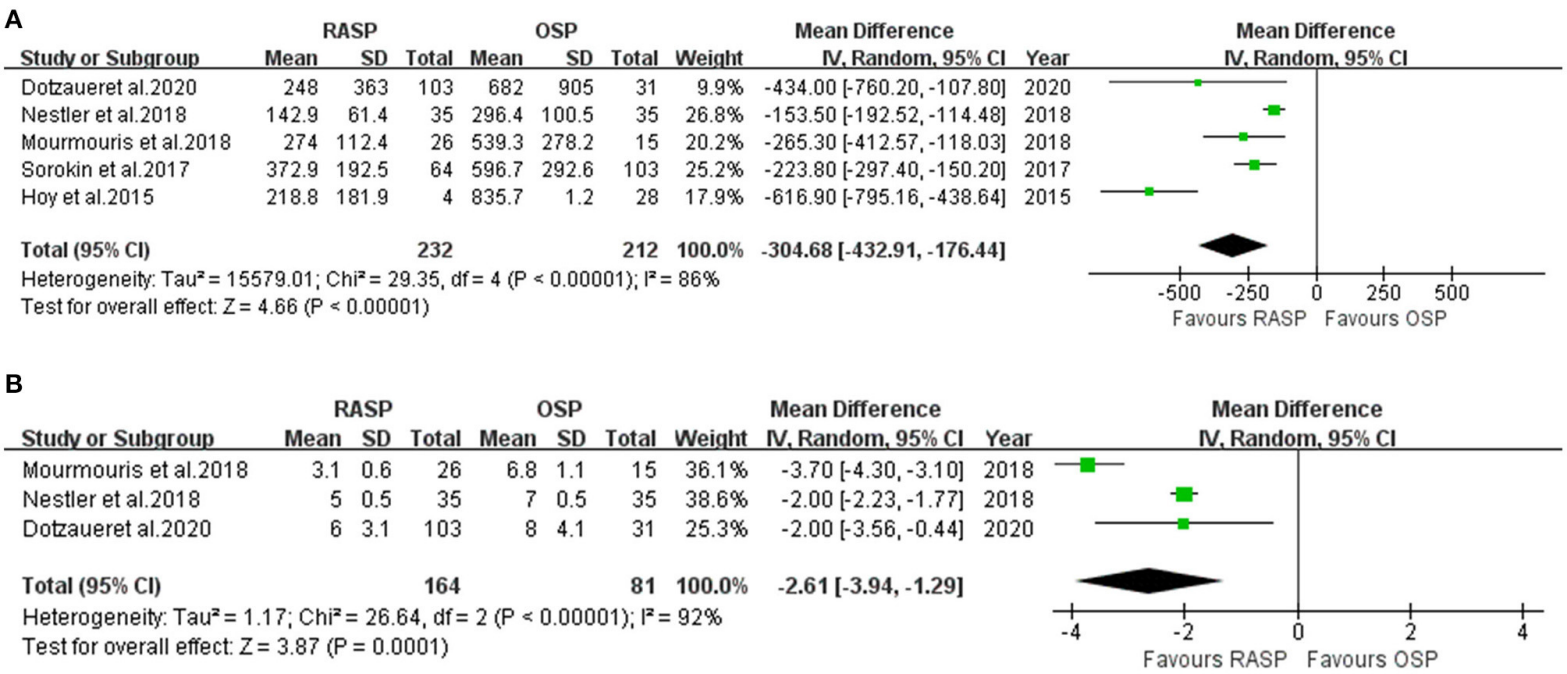
Forest plot and meta-analysis of EBL **(A)** and CT **(B)**.

Data on EBL were obtained from five studies (5, 10–13) including 444 patients (232 in the RASP group and 212 in the OSP group) (Figure 3A). The pooled outcome indicated that EBL was lower in the RASP group than in the OSP group (WMD, −304.68; 95% CI, −432.91, −176.44; *P* < 0.001; *I*^2^ = 86%). The difference in EBL was statistically significant between the RASP and OSP groups.

#### Catheterization Time

There were 245 patients analyzed in three studies (5, 10, 13). There was a statistically significant difference in CT between the two groups, with the CT in the RASP group being shorter than that in the OSP group (random-effects model: WWD, −2.61; 95% CI, −3.94, −1.29; *P* < 0.001; *I*^2^ = 92%), despite high heterogeneity (Figure 3B).

#### Complications

**Figure 4:**
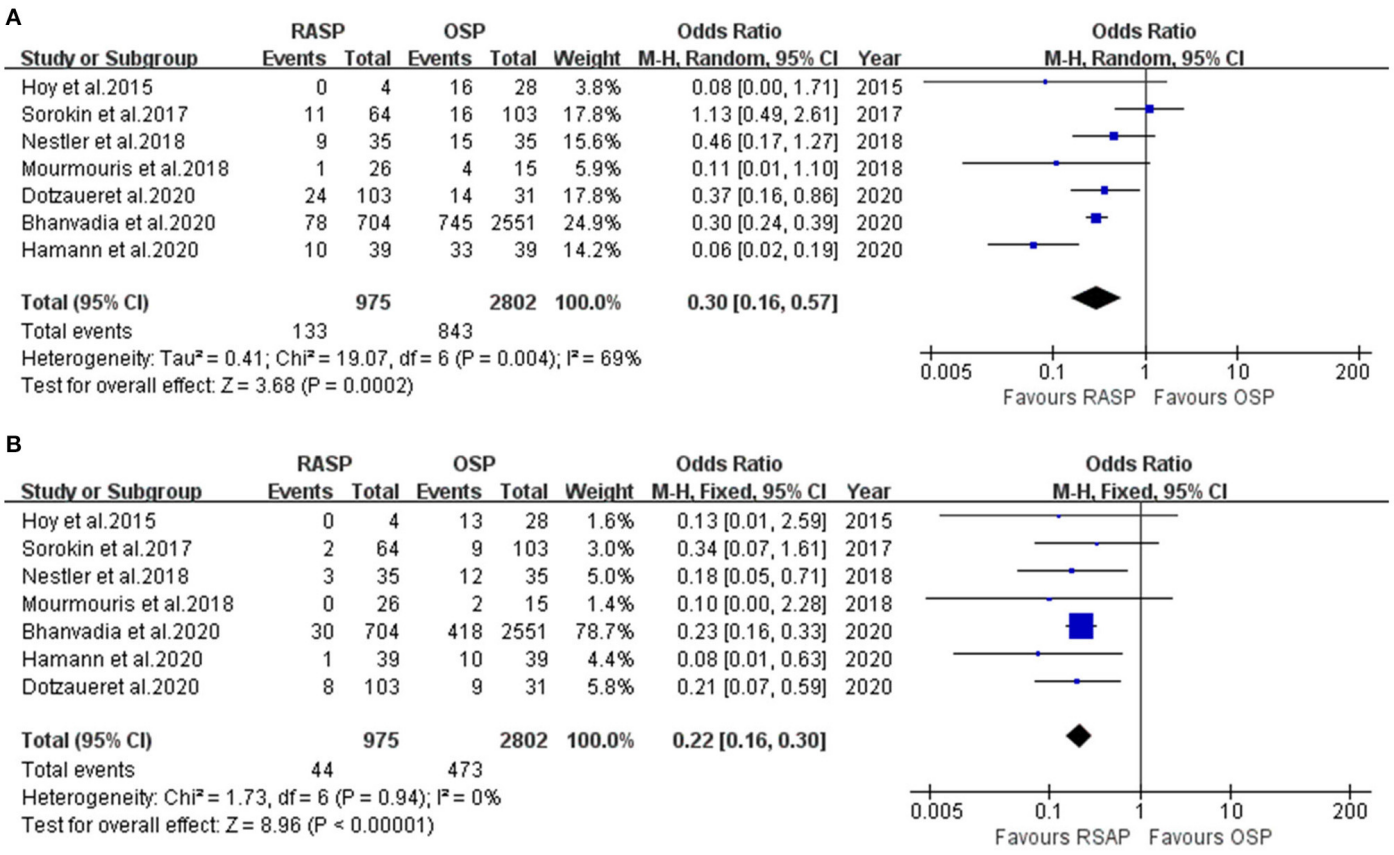
Forest plot and meta-analysis of TR **(A)** and complications **(B)**.

The RASP group had a significantly lower TR than the OSP group (OR, 0.22; 95% CI, 0.16, 0.30; *P* < 0.001; *I*^2^ = 0%; Figure 4B). The results were significantly different.

The forest plot in Figure 4A illustrates the complications of the RASP and OSP groups. There was a clinically meaningful difference between the two groups in terms of complications; RASP was associated with fewer complications in the total analysis (random-effects model: OR, 0.30; 95% CI, 0.16, 0.57; *P* < 0.001; *I*^2^ = 69%).

#### Evaluation of Efficacy

As shown in Table 3, there were no significant differences between the RASP and OSP groups in post-operative IPSS (WMD, 0.72; 95% CI, −0.31, 1.76; *P* = 0.17), QoL (WMD, 0.00; 95% CI: −0.39, 0.39; *P* > 0.99), Qmax (WMD, 1.88; 95% CI, −1.15, 4.91; *P* = 0.22), or PVR (WMD, −10.48; 95% CI, −25.13, 4.17; *P* = 0.16).

**Table 3:** Overall analysis of post-operative efficiency parameters comparing RASP and OSP.

**Results**	**No. of studies**	**Patient(*N*)**	***P*-value**	**WMD (95%CI)**
		**RASP vs. OSP**		
IPSS	2	90/118	0.17	0.72
Qmax	3	193/149	0.22	1.88
PVR	2	90/118	0.16	−10.48
QoL	1	64/103	1.00	0

*IPSS, International Prostate Symptom Score; QoL, quality of life; Qmax, maximum urinary flow rate; PVR, post-void residual urine volume*.

## Discussion

Current guidelines recommend OSP as the standard treatment for large prostate glands (≥80 g) (16, 17). Although OSP can significantly improve LUTS in patients with BPH, it is also associated with longer LOS, longer CT, and higher EBL than RASP, as well as a higher rate of complications (5, 10–13). Therefore, open prostatectomy is becoming less common. With the advent of the modern era of minimally invasive urology, multiple minimally invasive procedures have been used to treat large glands, including prostate laser enucleation, laparoscopic surgery, and RASP. Because of the longer learning curve, the incidence of post-operative urethral stricture, and the transient SUI associated with laser enucleation and the limited availability of laparoscopic prostatectomy, RASP is generally considered the optimal minimally invasive alternative, especially in the treatment of large glands. Some studies have indicated that RASP has significant advantages, such as less bleeding and lower surgical morbidity, and could be used to simultaneously treat bladder-related diseases (8, 18). Because the efficacy and safety of RASP and OSP in the treatment of large prostate glands remains debated, we conducted a meta-analysis to obtain systematic evidence.

We included seven studies involving 3,777 patients in our study and compared the efficacy and safety of RASP and OSP. This new meta-analysis showed that RASP and OSP had similar post-operative outcomes in terms of functional parameters, both subjective (IPSS and QoL) and objective (Qmax and PVR) variables. However, few studies assessed urination after these two surgeries. In recent years, only two studies Dotzauer et al. (5) and Sorokin et al. (12) reported no differences between RASP and OSP in terms of IPSS, QoL, Qmax, and PVR. However, these studies were retrospective and had a small number of cases and short follow-up time. Therefore, these results need to be interpreted with caution, and more large randomized controlled trials are needed to obtain better evidence.

Our data suggest that RASP had advantages over OSP, including less blood loss, shorter time to catheter removal, lower EBL, shorter LOS, less blood transfusion, and fewer complications. However, the results of our meta-analysis showed that RASP was associated with a significantly longer OT than OSP. This result is consistent with those in previous studies assessing robot-assisted prostatectomy (7, 19, 20). The duration of surgery seemed to be related to a higher BMI (5), applying the ports and docking the robot (13), and the surgeon's technical proficiency (21). However, there was high heterogeneity in these findings (*I*^2^ = 55%), which may be because BMI was examined in only two studies, and the patients in the two studies were different.

Blood loss is a major focus of surgical attention. EBL was assessed in all seven studies included in this analysis, and the results showed that RASP was associated with less blood loss than OSP. Correspondingly, the rate of post-operative blood transfusion was lower with RASP. Two recent studies strengthened the level of evidence in our research. Dotzauer et al. (5) compared the EBL and the rate of post-operative blood transfusion between OSP and RASP and found that both EBL (OSP vs. RASP: 682 ± 905 mL vs. 248 ± 363 mL, *P* = 0.007) and post-operative blood transfusion (OSP vs. RASP: 29 vs. 8%, *P* = 0.004) were lower in the RASP group. A multicenter study also showed that the TR in the RASP group was lower than that in the OSP group (3 vs. 26%, *P* < 0.05) (14). In contrast to our findings, Sorokin et al. (12) showed no significant difference in TR between the RASP and OSP groups in their propensity score-matched comparison. This discrepancy may be related to the contemporary guidelines by urologists and patient comorbidities (13). The lower EBL of RASP is associated with the 3D view, ergonomic comfort, better view of the surgical field, and high surgical precision.

Only three studies reported CT. The meta-analysis indicated lower CT for RASP than OSP with high heterogeneity, which is likely because extubation standards were different in different studies (22). However, Kordan et al. (23) conducted a systematic review and meta-analysis and found that many patients were discharged from the hospital with their foley catheters, and the catheters were removed later. Therefore, CT may not be a good indicator for evaluating RASP outcomes. Further research is needed to confirm these results.

Although increasing evidence comparing RASP and OSP has been compiled (5, 14, 15), only six studies examined the LOS. The results showed that RASP significantly reduced the LOS. This is in agreement with the results of recent studies, including two large studies that further validate our results (5, 14, 15, 22, 24). There are many factors that affect the LOS, including surgical experience, complications, indwelling CT, and ASA classification (5).

The incidence of complications is an important index to evaluate the safety of surgery. Our meta-analysis suggested that there was a significant difference in the incidence of overall complications in patients undergoing RASP and those undergoing OSP. RASP could effectively reduce the incidence of complications. Bhanvadia et al. (15) observed similar results using data from the Nationwide Inpatient Sample (NIS) from 2013 to 2016 (total complications, 11.1 vs. 29.2%, *P* < 0.01). This may be related to the following factors: (1) patients who undergo RASP are younger and have fewer comorbidities (15), and (2) different surgical approaches, i.e., OSP is performed retropubically, whereas a transperitoneal transvesical approach is chosen for RASP (5).

Finally, the cost of RASP has always been a concern, and whether RASP can be widely utilized, especially in developing countries, needs to be addressed. To our surprise, an online survey of urologists on treatment decisions for BPH found that doctors were mainly concerned about safety, effectiveness, and their own experience, rather than cost (25). Several studies have examined cost associated with RASP (3, 14, 25, 26). Sutherland et al. (3) found that the cost of RASP was twice that of OSP ($5,212 vs. $2,415). The NIS data also show a much higher total unadjusted hospitalization cost for RASP ($10,855 vs. $13,467, *P* < 0.01) (15). In contrast, Matei et al. (27) found that RASP cost €1,564 less on average than OSP, which may be related to faster convalescence and earlier return to robotic surgery. Bhanvadia et al. (15) used a large dataset and multivariate analysis to show that complications, LOS, and nursing facility care were related to the cost of RASP. However, cost is affected by multiple factors, and nursing care and earlier return to work cannot be easily quantified. Therefore, more large-scale research is needed to obtain better evidence.

As far as we know, this is the first meta-analysis that independently compares the efficacy and safety of RASP and OSP. However, there are some limitations in the analysis. First, only two prospective controlled study was included in our analysis, and most studies had a small sample size. Although one large-scale study was included, this study used NIS data, which has selection bias. Therefore, the level of evidence was reduced. Second, because of the heterogeneity of the studies examining LOS, complications, indwelling CT, and OT, the results need to be interpreted prudently. Furthermore, due to the small sample size, subgroup analysis was not possible. Third, few studies reported hospitalization costs, and there were many mixed factors, so it was difficult to obtain effective evidence. Finally, not all relevant studies could be detected by computer retrieval.

## Conclusion

The meta-analysis indicated that RASP is a viable and effective alternative to OSP. It provides similar functional outcomes and has better safety. Larger randomized control trials comparing RASP and OSP for large prostates may give us better evidence.

## Data Availability Statement

The original contributions generated for the study are included in the article/supplementary materials, further inquiries can be directed to the corresponding author.

## Author Contributions

ZX, JL, and XY: study design. HJ, CN, XL, YL, and ZZ: collection and analysis of data. ZX, JL, and JW: manuscript writing. All authors contributed to the article and approved the submitted version.

## Conflict of Interest

The authors declare that the research was conducted in the absence of any commercial or financial relationships that could be construed as a potential conflict of interest.
